# Allelic Analysis of *smc-6* Reveals Domain-Specific Roles in DNA Repair in *Caenorhabditis elegans*

**DOI:** 10.3390/ijms27114843

**Published:** 2026-05-27

**Authors:** Yiqiang Liang, Yingling Zhang, Junkai Xie, Qice Xiao, Guiyan Liao, Jie Lu

**Affiliations:** 1 College of Life Science and Technology, Guangxi University, Nanning 530004, China; 2 Institute of Biological Sciences and Technology, Guangxi Academy of Sciences, Nanning 530007, China

**Keywords:** SMC-5/6 complex, NSE-1, SMC-6, forward genetic screen, DNA repair, *Caenorhabditis elegans*

## Abstract

The Structural Maintenance of Chromosomes complex 5/6 (SMC-5/6) safeguards genome stability by coordinating DNA replication, repair, and chromosome organization. Although prior studies have advanced understanding of SMC-6, a domain-resolved view of its functions in vivo, particularly in multicellular organisms, remains incomplete. Because the non-SMC subunit NSE-1 localizes at the SMC-5/6 head interface and reflects complex integrity, we used NSE-1::GFP nuclear localization as a visual readout in an ethyl methanesulfonate (EMS)-based forward genetic screen in *Caenorhabditis elegans* (*C. elegans*). We identified three new *smc-6* alleles—*smc-6(wsh34)*, *smc-6(wsh35)*, and *smc-6(wsh36)* through single-nucleotide polymorphism (SNP) mapping and whole-genome sequencing. *smc-6(wsh34)* and *smc-6(wsh35)* affect the N-terminal ATPase domain, whereas *smc-6(wsh36)* lies in the hinge region. ATPase-domain mutants exhibited reduced fertility, decreased progeny viability, hypersensitivity to methyl methanesulfonate and cisplatin, and strong induction of the pro-apoptotic genes *egl-1* and *ced-13*. In contrast, the hinge mutant exhibited moderate fertility defects and partial sensitivity to DNA damage reagents. Structural modeling suggests that the R103 truncation disrupts the SMC-5/6 head interface, whereas the P514L substitution alters hinge dynamics. Together, these findings reveal a functional hierarchy in SMC-6, with the ATPase domain governing repair-associated energy-dependent processes and the hinge maintaining structural integrity.

## 1. Introduction

Structural maintenance of chromosomes (SMC) complexes are conserved ATPases that shape higher-order chromosome architecture and, in doing so, safeguard genome integrity [1,2]. Among these, the SMC-5/6 complex is particularly tightly coupled to replication-associated genome maintenance [3,4,5]. It has been implicated in double-strand break repair [6], stabilization and restart of perturbed replication forks, and the processing of recombination intermediates [4,6], thereby preventing deleterious rearrangements and replication collapse under genotoxic stress [4,5]. Consistent with these roles, compromised SMC-5/6 activity leads to genome instability and, in many organisms, to pronounced developmental and germline defects [7], underscoring a conserved requirement for SMC-5/6 in safeguarding reproductive genome integrity.

Within this complex, SMC-6 is a core ATPase subunit that, together with SMC-5, forms the structural backbone of the holocomplex [8]. Through its ATPase head and hinge regions, SMC-6 is thought to contribute to the conformational transitions, chromatin engagement, and DNA repair-associated transactions required for SMC-5/6 activity [6,9]. However, the in vivo consequences of distinct *smc-6* lesions remains insufficiently defined, particularly in multicellular organisms. *Caenorhabditis elegans* (*C. elegans*) provides a suitable model for addressing this question because of its short life cycle, tractable genetics, and well-defined germline make it possible to examine genome-maintenance defects at the reproductive, developmental, and molecular levels [10]. Previous studies have already connected the SMC-5/6 pathway to replication-associated genome stability and meiotic DNA repair in *C. elegans*, including genetic analysis of *smc-5* and *brc-1* interactions [11], as well as functional studies of NSE-4 [12], the NSE-3 homolog MAGE-1 [13], NSE-1 [14], and BRC-1/BRCA1 [15] in meiosis DNA repair. These findings provide a foundation for examining how different classes of *smc-6* mutations affect genome maintenance in vivo.

Because SMC-6 functions within the context of the SMC-5/6 holocomplex [8,9], analysis of *smc-6* in vivo benefits from a readout that reflects complex integrity and activity rather than only downstream phenotypic outcomes. Among the non-SMC elements, NSE-1 offers such an entry point [14,16,17]. NSE-1 was originally identified as an integral non-SMC subunit of the SMC-5/6 complex [16], and subsequent studies showed that it is not merely structural but also has important regulatory activity, including ubiquitin-ligase-associated function [17]. In *C. elegans*, NSE-1 has been shown to play a crucial role in meiosis and DNA repair [14]. In addition, the availability of an *nse-1::gfp* reporter enables direct visualization of NSE-1 nuclear localization in living animals. Using NSE-1 as a phenotypic readout therefore provides a practical means to identify mutations in *smc-6* that perturb the assembly, localization, or functional state of the SMC-5/6 complex.

This approach is valuable not only for defining the effects of *smc-6* mutations themselves, but also for understanding how perturbing a core SMC subunit influences the behavior of the entire SMC-5/6 complex in vivo [18,19]. Recent allele-based analysis of *smc-5* showed that lesions affecting different regions of the complex can produce separable genome-maintenance defects [18]. A comparable analysis of *smc-6* is therefore expected to help clarify how distinct alterations in a core subunit are translated into complex-level phenotypes. At the same time, different classes of *smc-6* mutations are unlikely to be mechanistically equivalent: early nonsense alleles may behave as severe loss-of-function variants, whereas missense substitutions may retain partial activity and reveal more specific structural or regulatory defects [18]. Systematic comparison of independently isolated *smc-6* alleles is thus needed to define how different SMC-6 lesions influence fertility, developmental progression, and responses to genotoxic stress [19,20], and in turn to refine our understanding of SMC-5/6 function in vivo.

In this study, we performed an ethyl methanesulfonate (EMS)-based forward genetic screen in *C. elegans* using *nse-1::gfp* as a visual reporter and isolated mutants with altered NSE-1 nuclear localization. we identified three new *smc-6* alleles combining SNP mapping and whole-genome sequencing. We then compared their effects on brood size, progeny viability, sensitivity to methyl methanesulfonate (MMS), hydroxyurea (HU), and cisplatin, developmental progression, and DNA damage-associated transcriptional responses. These analyses provide new genetic tools for investigating *smc-6* function and offer insight into how distinct *smc-6* lesions affect the in vivo activity of the SMC-5/6 complex in *C. elegans*.

## 2. Results

### 2.1. EMS Screen Identifies Mutants with Altered NSE-1::GFP Localization

**Figure 1:**
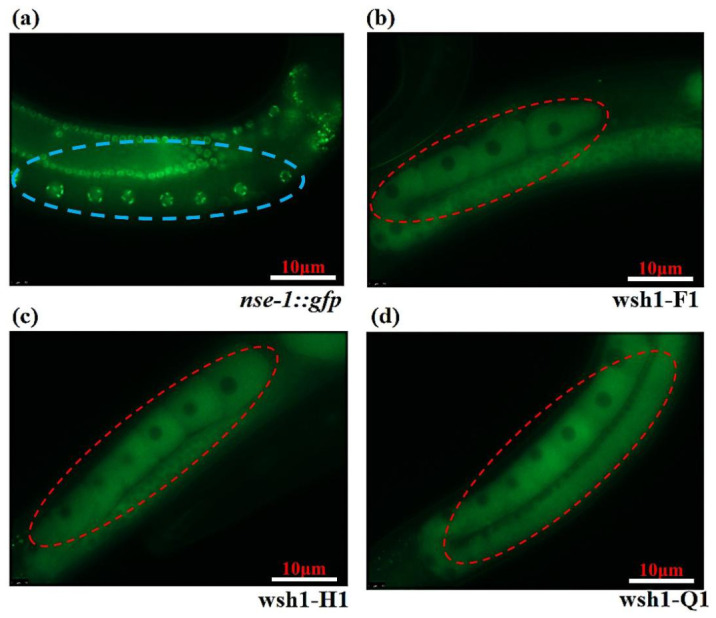
Isolation of mutants exhibiting aberrant NSE-1::GFP nuclear localization in the germline. Representative fluorescence micrographs of the *nse-1::gfp* reporter strain and three EMS-derived mutants selected for altered NSE-1::GFP distribution in gonadal cells. (**a**) In the control *nse-1::gfp* strain, NSE-1::GFP (green) shows robust enrichment within germline nuclei and a stereotyped, regularly patterned signal along the gonad (blue dashed outline). (**b**–**d**) In mutants *wsh1-F1* (**b**), *wsh1-H1* (**c**), and *wsh1-Q1* (**d**), NSE-1::GFP nuclear enrichment is markedly reduced and becomes diffusely distributed within the gonadal region (red dashed outlines), consistent with a reproducible mislocalization phenotype used for forward genetic screening. Images were acquired from stage-matched animals under identical acquisition settings to enable qualitative comparison across genotypes.

To dissect SMC-5/6 holocomplex integrity in vivo in an unbiased, phenotype-driven manner, we used germline NSE-1::GFP nuclear enrichment as a visual proxy for complex function and as the primary readout for an EMS forward genetic screen. In the *nse-1::gfp* control strain, NSE-1::GFP showed stable nuclear enrichment in gonadal nuclei, with strong, regularly patterned signals across chromosomal regions (Figure 1a), providing a reference pattern for normal localization during screening. Using this baseline, we screened approximately 26,600 EMS-mutagenized genomes and isolated 19 homozygous mutants with reproducible alterations in NSE-1::GFP distribution. Representative isolates are shown to illustrate the range of mislocalization phenotypes. Compared with the control, NSE-1::GFP nuclear enrichment was markedly altered in the gonads of mutants *wsh1-F1*, *wsh1-H1*, and *wsh1-Q1* (Figure 1b–d), characterized by reduced nuclear enrichment and a disorganized and diffuse intranuclear pattern. These consistent phenotypes provided a robust scoring criterion for mutant identification.

### 2.2. SNP Mapping Localizes All Three Independent Mutants to Linkage Group II (LGII)

**Figure 2:**
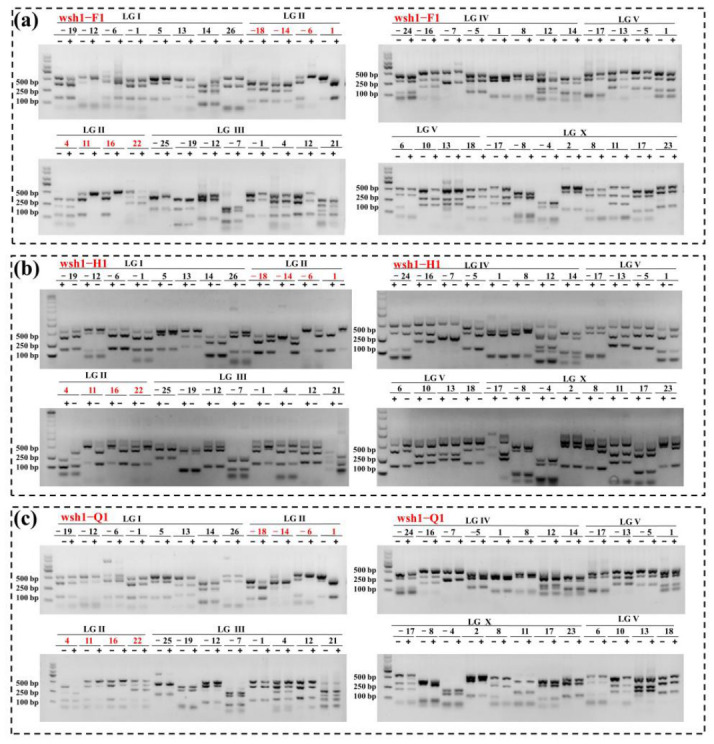
SNP-based chromosomal mapping localizes three independent NSE-1::GFP mislocalization mutants to LGII. Gel-based SNP mapping (PCR–RFLP) results for three phenotypically stable mutants: *wsh1-F1* (**a**), *wsh1-H1* (**b**), and *wsh1-Q1* (**c**). Each panel shows representative DraI-digested PCR products from a set of N2 Bristol vs. CB4856 (Hawaiian) polymorphic SNP markers distributed across the six linkage groups (LG I–V and LG X). Marker positions (genetic map coordinates, cM) are indicated above the corresponding lanes. For each mutant, animals displaying the NSE-1::GFP mislocalization phenotype and phenotypically normal controls were genotyped, and allele composition at each marker was inferred from the restriction fragment pattern after DraI digestion and agarose gel electrophoresis. Red numbers indicate informative LGII markers showing relative enrichment of the N2 (Bristol) allele in the mutant-phenotype pool and supporting the initial linkage assignment to chromosome II. All three mutants exhibit a consistent enrichment block on LGII (approximately −18 to +22 cM), whereas other linkage groups do not show comparably strong, contiguous enrichment, supporting that the causal lesions map to the same linked interval on chromosome II.

SNP-based mapping of three independent mutants (*wsh1-F1*, *wsh1-H1*, and *wsh1-Q1*) revealed a shared linkage signal. Across multiple adjacent SNP markers, mutant pools showed an overall enrichment trend of N2 (Bristol)-derived fragments relative to controls, consistent with linkage to the mislocalization phenotype. In all three cases, the peak enrichment localized to LGII (−18 to +22 cM), whereas other linkage groups lacked comparably strong, contiguous enrichment (Figure 2a–c). Together, these results indicate that the causal lesions in the three isolates likely map to the same linked interval.

### 2.3. Interval Mapping and WGS Identify smc-6 as the Causal Gene

**Figure 3:**
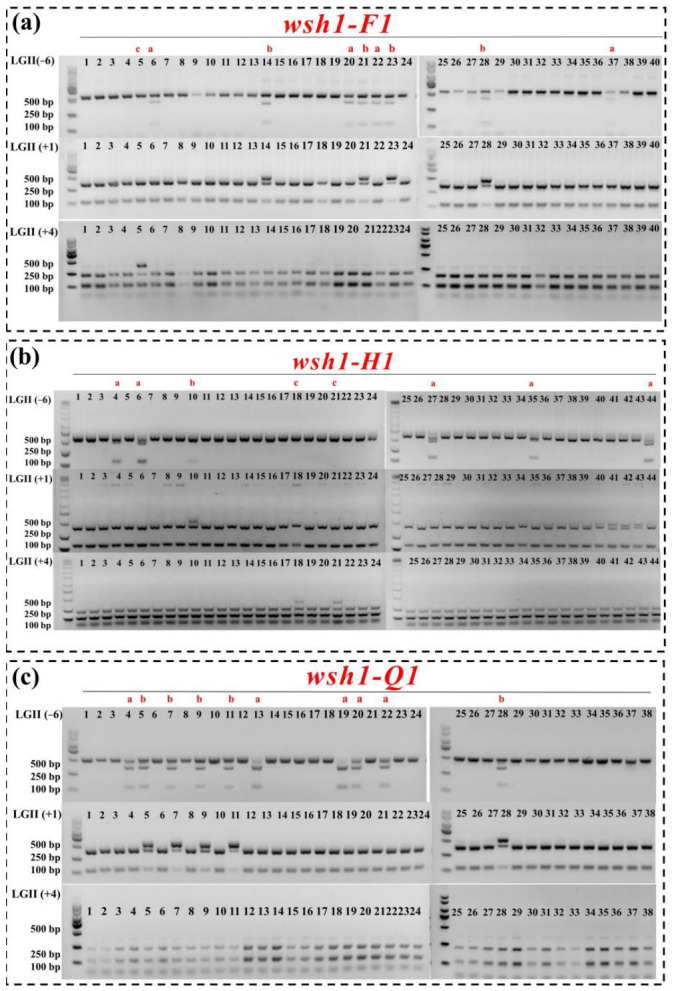
Gel-based SNP interval mapping (PCR–RFLP) for three independent NSE-1::GFP mislocalization mutants—*wsh1-F1* (**a**), *wsh1-H1* (**b**), and *wsh1-Q1* (**c**)—following crosses to CB4856 (Hawaiian). For each mutant, SNP markers spanning LGII were genotyped by PCR amplification, DraI restriction digestion, and agarose gel electrophoresis. The genetic positions (cM) of the assayed SNP markers are indicated above the corresponding lanes. Lowercase red letters above selected lanes denote distinct recombinant classes among independent interval-mapped lines that share the same SNP pattern across the informative markers; these labels do not indicate marker order or genetic distance.

After initially assigning the causal locus to LGII (−18 to +22 cM), we performed SNP interval mapping on *wsh1-F1*, *wsh1-H1*, and *wsh1-Q1* to increase mapping resolution. PCR–RFLP genotyping across LGII revealed highly concordant linkage among the three mutants, with the candidate region converging to a narrow interval of II:1–4 cM (Figure 3a–c). The recombinant distribution further supported this refinement. For *wsh1-F1*, 32 of 40 recombinants were homozygous for N2 (Bristol) DNA across the assayed SNP markers, whereas only a minority carried heterozygous calls or CB4856 (Hawaiian) segments at one or more positions, consistent with informative crossover events within the linked interval. A similar pattern was observed for *wsh1-H1* and *wsh1-Q1*, in which most recombinants carried Bristol alleles at the tested markers (36/44 and 28/38, respectively), with a small fraction showing recombinant genotypes. Together, these data indicate that the causal lesions in all three independent mutants map to the same sub-chromosomal interval on LGII.

**Figure 4:**
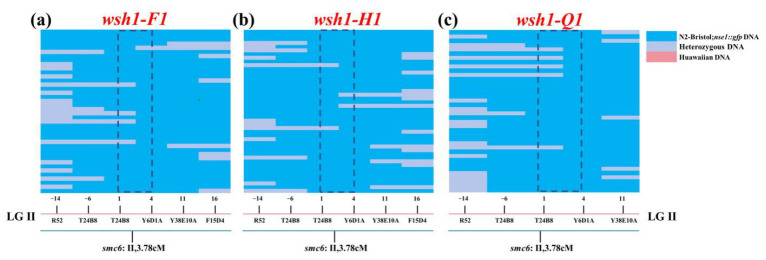
Recombinant-genotype maps summarizing interval mapping results for *wsh1-F1* (**a**), *wsh1-H1* (**b**), and *wsh1-Q1* (**c**). Each horizontal bar represents an individual recombinant line, and colors denote genotype at each marker (N2 (Bristol), heterozygous, or CB4856 (Hawaiian)). Vertical dashed boxes indicate the minimal shared interval refined by recombination, converging to II:1–4 cM. The number of recombinants analyzed, and the fraction retaining Bristol genotypes across the assayed markers are shown (*wsh1-F1*, 32/40; *wsh1-H1*, 36/44; *wsh1-Q1*, 28/38), supporting that all three mutants map to the same sub-chromosomal region on LGII.

We next integrated the refined mapping interval (II:1–4 cM) with whole-genome sequencing (WGS) variant calling to prioritize candidate mutations within the linked region, and summarized the results in recombinant/SNP mapping plots (Figure 4a–c). Combining interval mapping with WGS-based variant annotation ultimately identified smc-6 as the most likely causal gene underlying NSE-1::GFP nuclear mislocalization in *wsh1-F1*, *wsh1-H1*, and *wsh1-Q1*, providing the genetic foundation for subsequent analyses of *smc-6* alleles and their functional consequences.

### 2.4. Molecular Definition and Domain Mapping of smc-6 Mutants

**Figure 5:**
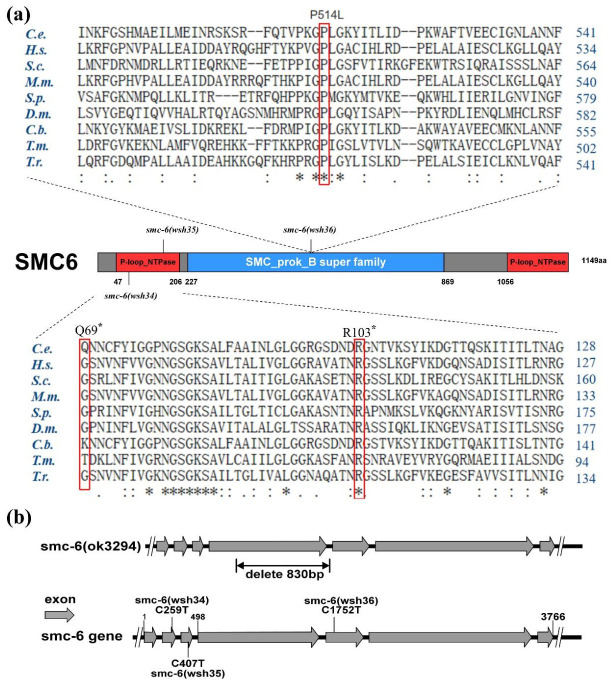
Molecular identification and domain mapping of *smc-6* alleles. (**a**) SMC-6 domain architecture and evolutionary conservation of residues affected by the newly isolated alleles. The positions of the ATPase-head truncations (Q69* and R103*) and the hinge-region substitution (P514L) are indicated on the linear SMC-6 schematic. Multiple-sequence alignments across representative species (*C. elegans, C.e*; *Homo sapiens*, *H.s*; *Saccharomyces cerevisiae*, *S.c*; *Mus musculus*, *M.m*; *Schizosaccharomyces pombe*, *S.p*; *Drosophila melanogaster*, *D.m*; *Caenorhabditis briggsae*, *C.b*; *Trypanosoma musculi*, *T.m*; *Takifugu rubripes*, *T.r*) highlight strong conservation of the mutated residues (boxed), supporting their functional importance. In the multiple-sequence alignments, the symbols below the sequences indicate the conservation level: “*” indicates fully conserved residues, “:“ indicates residues with strongly similar properties, and “.” indicates residues with weakly similar properties. (**b**) Gene structure of *smc-6* and molecular lesions. The reference loss-of-function allele *smc-6(ok3294)* carries an 830 bp deletion near the end of exon 4, predicted to cause a frameshift and premature termination (**top**). Whole-genome sequencing identified single-nucleotide substitutions in the three screen-derived alleles (**bottom**): *smc-6(wsh34)* (C259T; Q69*), *smc-6(wsh35)* (C407T; R103*), and *smc-6(wsh36)* (C1752T; P514L).

To define the molecular features of the three forward-screen isolates at the allelic level and to establish a comparative framework against a known loss-of-function allele, we renamed *wsh1-F1*, *wsh1-H1*, and *wsh1-Q1* as *smc-6(wsh34)*, *smc-6(wsh35)*, and *smc-6(wsh36)*, respectively. As a positive reference, we analyzed the laboratory deletion allele *smc-6(ok3294)*, which carries an 830 bp deletion near the end of exon 4, resulting in a frameshift after amino acid 213 and premature termination (Figure 5b). Using WGS alignments together with gene-model annotation, we confirmed that all three newly isolated mutants harbor single-nucleotide substitutions: C259T in *smc-6(wsh34)*, C407T in *smc-6(wsh35)*, and C1752T in *smc-6(wsh36)* (Figure 5b). To infer the functional consequences of these lesions, we mapped each mutation onto annotated SMC-6 domains and evaluated evolutionary conservation by multi-species sequence alignment. SMC-6 is highly conserved, with key functional modules including the P-loop NTPase (ATPase head) domain required for ATP binding/hydrolysis and additional conserved regions such as the SMC-prok-B domain (Figure 5a). Both *smc-6(wsh34)* and *smc-6(wsh35)* are nonsense alleles predicted to truncate the protein. Specifically, *smc-6(wsh34)* introduces a stop codon at residue 69, upstream of the Walker A motif, and thus eliminates an intact Walker A element; *smc-6(wsh35)* terminates at residue 103, within the critical segment between the Walker A and Walker B motifs. These truncations are expected to compromise ATPase-domain integrity and thereby abrogate core SMC-5/6 functions. In contrast, *smc-6(wsh36)* is a missense allele in which C1752T substitutes a highly conserved proline with leucine (P514L) within the hinge region. Given the strong conservation of this site across species (Figure 5a), the P514L change is likely to perturb hinge conformational properties and reduce, rather than eliminate, SMC-6 function. Collectively, this molecular stratification provides a structural basis for interpreting allele-specific phenotypic differences in subsequent analyses.

### 2.5. Baseline Fertility Defects in smc-6 Mutants

**Figure 6:**
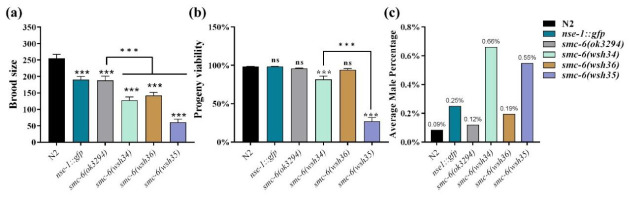
Baseline reproductive phenotypes of *smc-6* alleles (**a**–**c**) Baseline reproductive phenotypes under standard culture conditions (20 °C): brood size (**a**), progeny viability/hatching rate (**b**), and male frequency among surviving progeny (**c**) for the indicated genotypes (including N2, *nse-1::gfp* control, and *smc-6* alleles). L4 hermaphrodites were assayed in a single-worm-per-plate design with periodic transfers, and eggs/hatched larvae and males were scored as described in Methods. Bars represent mean ± SEM from at least three independent biological replicates (25 animals per genotype per replicate). Statistical significance was determined using one-way ANOVA with Tukey’s multiple-comparisons test; significance levels are indicated in the graphs (ns, not significant; ***: *p* < 0.001).

We quantified baseline reproductive phenotypes in the absence of exogenous DNA damage to assess how distinct *smc-6* alleles affect reproductive capacity, using *nse-1::gfp* as the negative control and the deletion allele *smc-6(ok3294)* as a positive control. Brood size was scored in a single-worm-per-plate assay for *smc-6(wsh34)*, *smc-6(wsh35)*, and *smc-6(wsh36)* (n = 25 hermaphrodites per genotype), with mothers transferred every 12 h and total egg production accumulated across the reproductive period. Compared with the control, all three point mutants exhibited significantly reduced brood sizes, indicating that compromised *smc-6* function directly limits reproductive output. Progeny viability was also decreased in *smc-6(wsh34)* and *smc-6(wsh35)*, with the strongest reduction observed in *smc-6(wsh35)*, whereas *smc-6(wsh36)* showed a comparatively mild effect on this metric. In addition, male frequency was elevated in *smc-6(wsh34)* and *smc-6(wsh35)*, consistent with increased X-chromosome nondisjunction and/or impaired chromosome stability during meiosis. Together, these results demonstrate allele-dependent reproductive defects in *smc-6* mutants, with lesions in key functional regions producing more severe baseline phenotypes (Figure 6a–c).

### 2.6. Reproductive Sensitivity of smc-6 Mutants to DNA-Damaging Agents

We systematically quantified reproductive sensitivity of three *smc-6* point alleles following treatment with an alkylating agent (MMS), a replication-stress inducer (HU), or an interstrand crosslinking agent (cisplatin) to delineate the contribution of SMC-6 to the repair of distinct classes of DNA lesions. Progeny viability (hatching rate) was used as the primary endpoint. The assay panel included wild-type N2 as a negative control, the *smc-6* deletion allele *smc-6(ok3294)* as an internal loss-of-function benchmark, and pathway reference mutants representing key repair modules, including *mus-81(tm1937)* (structure-specific endonuclease involved in replication-intermediate processing) [21], *brc-1(tm1145)* (homologous recombination–associated functions), and *lig-4(ok716)* (non-homologous end joining), enabling pathway-informed phenotypic comparisons.

**Figure 7:**
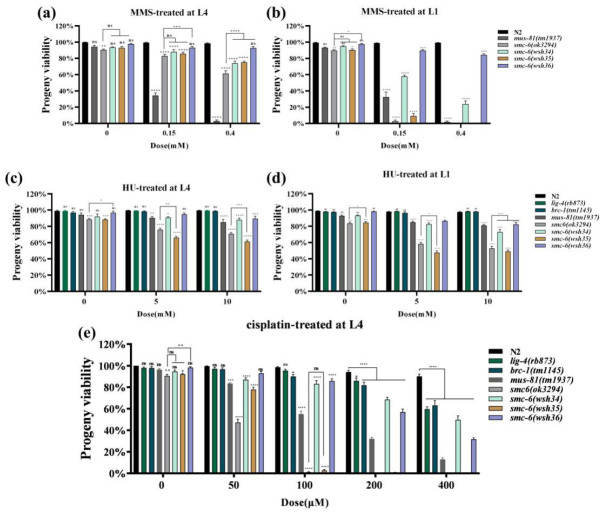
*smc-6* alleles exhibit dose- and stage-dependent hypersensitivity to genotoxic stress. Progeny viability was measured after exposing stage-matched animals to the indicated DNA-damaging agents, followed by washout and recovery. Genotypes include wild-type N2, the *nse-1::gfp* reporter control, *smc-6* alleles: *smc-6(ok3294)*, *smc-6(wsh34)*, *smc-6(wsh35)*, *smc-6(wsh36)*, and pathway reference mutants: *mus-81(tm1937)*, *brc-1(tm1145)*, *lig-4(ok716)* as indicated in the legends. (**a**,**b**) MMS treatment at the L4 stage (**a**) or L1 stage (**b**) at 0, 0.15, and 0.4 mM. (**c**,**d**) HU treatment at the L4 stage (**c**) or L1 stage (**d**) at 0, 5, and 10 mM. (**e**) Cisplatin treatment at the L4 stage at 0, 50, 100, 200, and 400 μM. Bars show mean ± SEM from at least three independent biological replicates. Statistical significance is indicated above comparisons (one-way ANOVA with Tukey’s multiple-comparisons test; ns, not significant; * *p* < 0.05; ** *p* < 0.01; *** *p* < 0.001; **** *p* < 0.0001).

MMS exposure caused a pronounced, dose-dependent reduction in progeny viability across all *smc-6* mutants, supporting an essential role for SMC-6 in tolerating alkylation-induced damage (Figure 7a,b; L4- and L1-stage treatments, respectively). Among the alleles, the ATPase-head truncation *smc-6(wsh35)* phenocopied the severe sensitivity of *smc-6(ok3294)* and closely resembled *mus-81(tm1937)*, consistent with the notion that disrupting the SMC-6 ATPase module produces a defect comparable in magnitude to loss of a core replication-intermediate processing factor. The early N-terminal nonsense allele *smc-6(wsh34)*, which introduces a premature stop codon upstream of the Walker A motif and is therefore predicted to eliminate an intact Walker A element, generally showed an intermediate phenotype: this lesion would be expected to impair ATP binding/hydrolysis and coordinated head engagement, but its phenotypic severity remained consistently lower than that of *smc-6(wsh35)*, suggesting that its functional consequences are substantial but less severe in these assays. In contrast, the hinge-region missense allele *smc-6(wsh36)* (P514L) exhibited a markedly attenuated decrease in viability, indicating partial functional retention under MMS challenge. HU treatment revealed clearer allele-specific separation. Progeny viability was strongly compromised in *smc-6(wsh35)* and *smc-6(ok3294)*, whereas *smc-6(wsh34)* showed a comparatively mild effect and, like *smc-6(wsh36)*, showed little to no reduction relative to controls across the tested conditions (Figure 7c,d; L4- and L1-stage treatments, respectively). These results underscore the requirement of an intact SMC-6 ATPase module for coping with replication stress, while suggesting that the hinge lesion confers a comparatively mild defect in this context. Cisplatin exposure produced the most striking phenotypic stratification. *smc-6(wsh35)* and *smc-6(ok3294)* were extremely hypersensitive, with dose–response profiles closely tracking that of *mus-81(tm1937)* and exhibiting substantially greater sensitivity than *brc-1(tm1145)* or *lig-4(ok716)* (Figure 7e). *smc-6(wsh34)* also showed clear cisplatin sensitivity, although its defect remained less severe than that of *smc-6(wsh35)*. *smc-6(wsh36)* displayed reduced viability only at higher doses and remained clearly less affected than *smc-6(wsh35)*, again consistent with a hypomorphic effect of the hinge mutation.

Collectively, these reproductive sensitivity assays demonstrate that SMC-6 is critical for resisting MMS- and cisplatin-induced complex lesions and support a domain hierarchy in which ATPase-head integrity is indispensable for robust repair/tolerance, whereas a single conserved hinge substitution produces a partial-loss phenotype.

### 2.7. Genotoxin-Induced Developmental Delay in smc-6 Mutants

**Figure 8:**
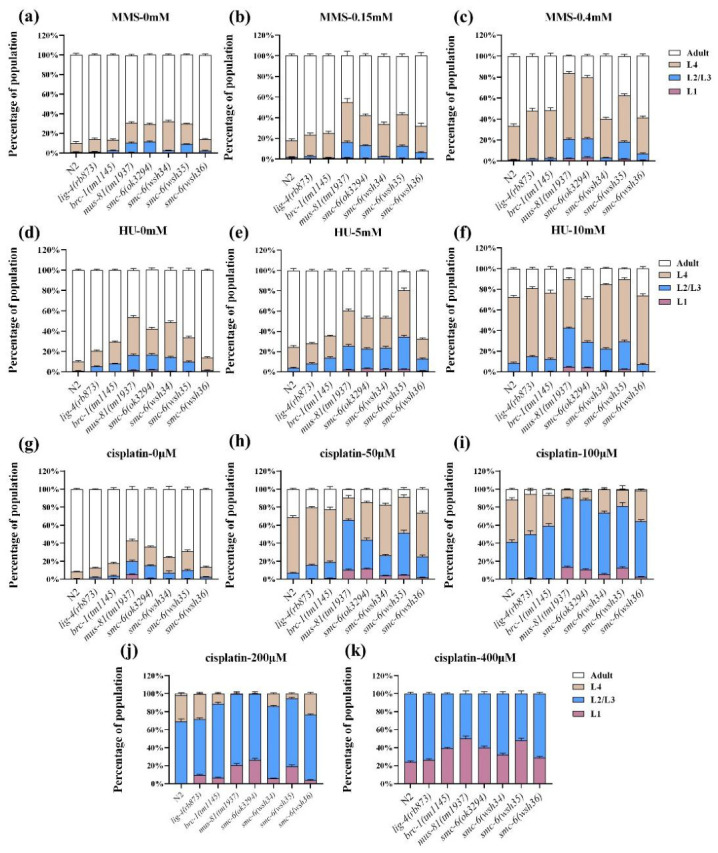
Genotoxic stress induces dose-dependent developmental delay in *smc-6* mutants. Stacked bar plots show the distribution of developmental stages in populations derived from synchronized L1 larvae after exposure to DNA-damaging agents and subsequent culture for 48 h, scored as the percentage of animals at L1 (pink), L2/L3 (blue), L4 (tan), or adult (white). Genotypes are indicated on the *X*-axis (N2; *lig-4(ok716)*; *brc-1(tm1145)*; *mus-81(tm1937)*; *smc-6(ok3294)*; *smc-6(wsh34)*; *smc-6(wsh35)*; *smc-6(wsh36)*). (**a**–**c**) MMS treatment at 0 mM (**a**), 0.15 mM (**b**), and 0.4 mM (c**).** (**d**–**f**) HU treatment at 0 mM (**d**), 5 mM (**e**), and 10 mM (**f**). (**g**–**k**) Cisplatin treatment at 0 μM (**g**), 50 μM (**h**), 100 μM (**i**), 200 μM (**j**), and 400 μM (**k**). Bars represent mean percentages, with error bars indicating SEM across independent biological replicates, as described in Methods.

To assess the impact of DNA damage on somatic developmental progression, synchronized L1 larvae were exposed to the indicated genotoxic agents and, after a 48 h recovery period, the population was scored for the proportion of animals at each developmental stage (L1–L4 and adult; Figure 8). Across MMS, HU, and cisplatin treatments, *smc-6* mutants displayed developmental delay to varying extents, manifested as a dose-dependent shift of the population toward earlier larval stages relative to wild type (Figure 8a–k). Under MMS exposure (0, 0.15, and 0.4 mM; Figure 8a–c), the ATPase-domain–disrupting alleles *smc-6(wsh34)* and *smc-6(wsh35)*, as well as the deletion allele *smc-6(ok3294)*, showed the strongest developmental arrest. Notably, *mus-81(tm1937)* exhibited the most severe arrest following MMS treatment, consistent with its pronounced sensitivity in progeny viability assays. HU treatment (0, 5, and 10 mM; Figure 8d–f) further highlighted allele-dependent differences: ATPase-compromised alleles and *smc-6(ok3294)* accumulated at larval stages at moderate to high doses, whereas the hinge missense allele *smc-6(wsh36)* displayed a comparatively mild delay. Cisplatin treatment (0, 50, 100, 200, and 400 μM; Figure 8g–k) similarly produced a clear dose-dependent stratification, with ATPase lesions and the deletion allele showing a marked reduction in adult fraction and increased retention in early larval stages at higher doses. Importantly, even in the absence of exogenous treatment (0-dose controls), some *smc-6* mutants showed a modest tendency toward delayed development, suggesting a baseline impairment in developmental robustness. Together, these results indicate that SMC-6 function is required not only for germline-associated outcomes but also for maintaining somatic developmental resilience under genotoxic stress, supporting a broad role for SMC-6 in organismal genome maintenance.

### 2.8. RT–qPCR Analysis of Pro-Apoptotic Gene Expression in smc-6 Mutants

**Figure 9:**
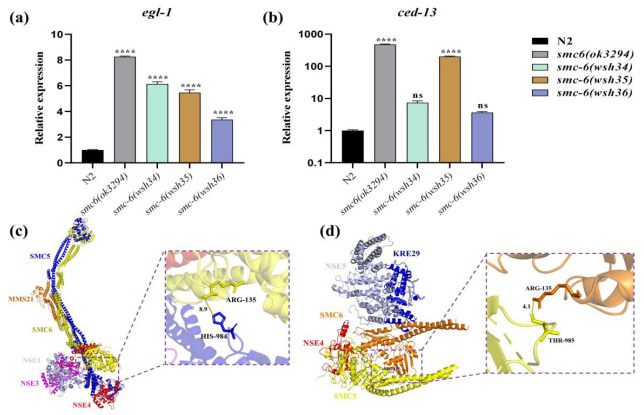
ATPase-defective *smc-6* alleles activate CEP-1/p53-dependent pro-apoptotic transcription and structural modeling places the corresponding residue at the SMC5–SMC6 head interface. RT–qPCR analysis of the CEP-1/p53 target genes *egl-1* (**a**) and *ced-13* (**b**) in stage-matched animals of the indicated genotypes (N2, *smc-6(ok3294)*, *smc-6(wsh34)*, *smc-6(wsh35)*, and *smc-6(wsh36)*). Expression is shown relative to N2 (set to 1). Bars represent mean ± SEM from independent biological replicates; statistical significance is indicated above bars (ns, not significant; **** *p* < 0.0001). Structural context of the *smc-6(wsh35)* lesion inferred by homology modeling based on the budding yeast SMC-5/6 cryo-EM architecture. (**c**) Overview of the SMC-5/6 complex with associated NSE subunits; inset highlights the position of Smc6 Arg-135 (corresponding to *C. elegans* SMC-6 R103) near a neighboring residue (His-984) in the head-region environment (distance shown). (**d**) Model of the assembled holocomplex (subunits labeled as indicated); inset shows Smc6 Arg-135 positioned in close proximity to Smc5 Thr-985 (~4.1 Å), consistent with a potential inter-subunit stabilizing interaction at the SMC5–SMC6 head interface.

To assess activation of apoptosis-related transcription downstream of DNA damage signaling in *smc-6* mutants, we quantified transcript levels of two core pro-apoptotic genes *egl-1* and *ced-13* by RT–qPCR (Figure 9a,b). Both genes are canonical transcriptional targets of the C. elegans p53 ortholog CEP-1, and their induction provides a molecular signature of CEP-1/p53-dependent DNA damage signaling and pro-apoptotic transcription [22]. In ATPase-compromised alleles, *smc-6(ok3294)* and *smc-6(wsh35)*, *egl-1* and *ced-13* were robustly upregulated, with particularly strong induction of *ced-13* and the largest overall response in *smc-6(wsh35)*. In contrast, the hinge missense allele *smc-6(wsh36)* showed only modest changes, with no significant difference from wild type. Notably, the N-terminal truncation allele *smc-6(wsh34)* displayed a mixed pattern: *egl-1* was significantly induced, whereas *ced-13* showed no significant change, suggesting that individual CEP-1 targets may respond differentially to distinct lesion types or repair defects. Together, these results provide molecular evidence that loss of SMC-6 function, particularly disruption of the ATPase module, is associated with elevated germline DNA damage signaling and induction of CEP-1/p53-dependent pro-apoptotic transcription, which may contribute to reduced fertility and compromised progeny viability.

### 2.9. Structural Modeling of Key SMC-6 Lesions

To rationalize allele severity at the structural level, we performed homology-based modeling guided by the cryo-EM architecture of the budding yeast SMC-5/6 complex. For the *smc-6(wsh35)* nonsense lesion at R103 (corresponding to Smc6 R135 in yeast), the model places this residue near the heterodimeric head interface formed by SMC5 and SMC6 (Figure 9c,d). In the assembled holocomplex, SMC6 R135 lies in close proximity to a neighboring SMC5 residue (e.g., T985), with an inter-residue distance of ~4.1 Å (Figure 9d), compatible with electrostatic and/or hydrogen-bonding interactions that could stabilize the head interface and support conformational coupling. Premature termination at R103 is therefore expected to disrupt this structural hub, impair complex assembly and compromise coordinated ATPase-head cycling, providing a structural explanation for why *smc-6(wsh35)* approaches the severity of the deletion allele *smc-6*(*ok3294)*. By contrast, the hinge missense substitution P514L in *smc-6(wsh36)* affects a highly conserved position within the hinge core; replacing proline is expected to perturb local backbone rigidity and hinge dynamics, consistent with a hypomorphic rather than null phenotype. Collectively, the structural analysis supports a domain hierarchy in which disruption of the head interface or ATPase module yields catastrophic loss of function and strong apoptotic activation, whereas hinge perturbation introduces a more moderate regulatory defect.

## 3. Discussion

In this study, we established a visualization-based forward genetic screening platform in *C. elegans*, using nuclear enrichment of NSE-1::GFP in the germline as an in vivo readout of SMC-5/6 holocomplex integrity and functional state. Screening ~26,600 EMS-mutagenized genomes yielded multiple stable mutant lines with reproducible NSE-1::GFP mislocalization, and three independent isolates were ultimately mapped to *smc-6*, thereby generating an *smc-6* allelic series suitable for structure–function analysis. By integrating SNP mapping, whole-genome resequencing, and targeted validation, we identified two nonsense lesions predicted to disrupt the ATPase head (*wsh34* and *wsh35*) and one missense substitution in the hinge region (*wsh36*). These mutations affect conserved residues critical for SMC architecture and produced consistent, graded phenotypic differences across fertility, progeny viability, genotoxin sensitivity, developmental progression, and apoptosis-related outputs. In *C. elegans*, previous studies on *smc-5*, NSE-1, NSE-4, MAGE-1/NSE-3, and BRC-1/SMC-5/6 interactions have already established important roles for this pathway in genome stability, DNA repair, and meiosis [11,12,13,14,15,18]. Together, the data support a domain hierarchy model in which the N-terminal ATPase head of SMC-6 is indispensable for robust genome maintenance, whereas the hinge primarily contributes to structural flexibility and fine-tuning of complex function.

A major advance of this work is methodological. NSE-1 localization provides an information-rich proxy for SMC-5/6 functional state that can be read directly in vivo. In the parental reporter strain, NSE-1::GFP is stably enriched in germline nuclei, establishing a reliable baseline for scoring mislocalization under standardized imaging conditions. In budding yeast, Nse1 has been characterized as an integral non-SMC subunit of the SMC-5/6 complex and as part of the Nse1–Nse3–Nse4 subcomplex, with important structural and regulatory functions [16,17,23]. In *C. elegans*, NSE-1 has been shown to play a crucial role in meiosis and DNA repair [14]. This strategy is therefore consistent with evidence from both yeast and nematode systems that perturbation of core SMC-5/6 architecture can be reflected in NSE-1 behavior. Notably, recent allele-series analyses of *smc-5* in *C. elegans* suggested that subtle disruption at the head–kleisin connection can propagate to NSE-1 mislocalization, implicating defects in the chromosomal assembly of the holocomplex [18]. Our findings extend this framework to SMC-6: monitoring NSE-1::GFP localization effectively captures lesions in both the ATPase and hinge modules, highlighting its sensitivity and breadth as an entry point for genetic discovery and functional stratification.

The molecular nature of the three *smc-6* alleles provides a direct explanation for phenotypic stratification. *wsh34* and *wsh35* introduce premature termination within the N-terminal ATPase head, and are expected to compromise the Walker motif framework required for ATP binding/hydrolysis as well as the coordinated head engagement mechanism. Such truncations are likely to block the ATPase-driven conformational cycle [24], consistent with severe fertility defects, pronounced hypersensitivity to genotoxic agents, and strong developmental delay. In contrast, *wsh36* carries a P514L substitution at a highly conserved hinge residue. Given the strong evolutionary conservation of this position, it is more likely to support hinge geometry and opening–closing dynamics rather than directly abolish catalytic activity. Accordingly, *wsh36* displays intermediate phenotypes and partial stress sensitivity, consistent with a hypomorphic allele: core assembly and basal function may be retained, while efficiency or regulatory precision is reduced.

Across lesion types, we observed a clear functional hierarchy between ATPase-head and hinge mutations. Cisplatin (interstrand crosslinks) most strongly separated allele severity: the severe ATPase allele *wsh35* closely resembled the strong loss-of-function background *ok3294* and exhibited dose–response profiles overlapping those of the replication intermediate processing mutant *mus-81*, whereas *wsh36* became overtly sensitive only at higher cisplatin doses. These patterns suggest that, under highly toxic replication-blocking lesions, ATPase-driven conformational switching of SMC-6 may be essential for stabilizing, remodeling, or processing stalled replication intermediates [3,5,25]. Consistently, after MMS, HU, or cisplatin exposure of synchronized L1 larvae, *smc-6* mutants accumulated at larval stages in a dose-dependent manner, with the most pronounced arrest in ATPase-compromised alleles, indicating that genome maintenance defects under replication stress can be amplified into organismal developmental fragility [20].

We further linked genome instability to reproductive defects through apoptosis-associated molecular readouts. RT–qPCR revealed robust induction of the CEP-1/p53 target genes *egl-1* and *ced-13* in severe backgrounds (*ok3294* and *wsh35*), whereas induction in the hinge allele *wsh36* was weak or not significant. Notably, *wsh34* exhibited a mixed pattern (*egl-1* induced, *ced-13* unchanged), suggesting that distinct alleles may engage different branches or activation thresholds within the germline damage response. Together with reduced fertility and hatching rates, these data support a model in which persistent DNA damage elevates checkpoint and apoptotic pressure [26], reducing the pool of functional germ cells and compromising embryo viability. In this sense, the allelic series helps distinguish catastrophic loss of repair capacity (ATPase truncations) from structural or regulatory perturbations (hinge missense), the latter permitting viability but lowering robustness under stress.

Structural modeling provides a physical framework for these domain-dependent outcomes. Homology modeling guided by the budding-yeast SMC-5/6 cryo-EM architecture places the residue corresponding to *wsh35* at or near the SMC5–SMC6 head dimer interface [24], a key node that couples ATP binding/hydrolysis to head engagement and higher-order conformational organization of the holocomplex [27]. Thus, truncation of the head module is expected to destabilize interface integrity and/or impair proper assembly and productive chromatin-bound conformations. In contrast, P514L is more plausibly modeled as a subtle mechanical perturbation of hinge properties [28,29]—altering local backbone constraints and steric environment—thereby affecting conformational flexibility without necessarily abolishing global folding or head function. Within this structural framework, the yeast-based model provides a useful reference for interpreting the positions of the corresponding *C. elegans* lesions and their associated in vivo phenotypes. Integrating localization, genetics, and structural information, we propose a working model in which ATPase-head integrity is required to maintain or transition into effective chromatin-engaged repair states, and its disruption leads to broad replication-associated sensitivity and strong apoptotic activation; hinge perturbation primarily reduces the efficiency of conformational transitions and/or chromatin interactions, producing a hypomorphic phenotypic spectrum.

Several limitations remain and motivate future work. First, although mapping and sequencing strongly implicate *smc-6*, definitive causality can be further established by transgenic rescue or precise CRISPR recreation of each allele in a clean background. Second, our inference of holocomplex integrity largely derives from localization and organism-level phenotypes; direct measurements of complex assembly/stability, chromatin-binding dynamics, and ATPase-dependent conformational cycling (e.g., co-immunoprecipitation, fractionation, live-imaging recovery assays, or ChIP occupancy) will be necessary to test the proposed model. Third, positioning SMC-6 within repair pathway networks will require systematic genetic interaction analyses with *brc-1*, *lig-4*, *mus-81* and related backgrounds to resolve upstream/downstream or parallel relationships under distinct lesion contexts. Finally, continued identification of the remaining non–*smc-6* mutants recovered from the NSE-1::GFP screen may uncover additional regulatory nodes (e.g., SUMO pathway factors or kleisin/interface components), expanding our understanding of SMC-5/6 assembly and regulation in vivo.

In summary, we establish NSE-1::GFP nuclear localization as a practical in vivo indicator of SMC-5/6 integrity in *C. elegans* and generate a new *smc-6* allelic series that reveals a clear division of labor and hierarchy between ATPase-head–mediated core functions and hinge-mediated structural regulation. Together with previous *C. elegans* studies on the SMC-5/6 pathway [11,12,13,14,15,18], this genetic toolkit and the domain hierarchy model provide a foundation for dissecting how SMC-5/6 coordinates replication stress responses, lesion processing, and germline genome protection in the nematode.

## 4. Materials and Methods

### 4.1. Reagents and Instruments

NGM plates and M9 buffer were prepared according to standard *C. elegans* procedures [10]. EMS, MMS, HU, and cisplatin were purchased from Sigma-Aldrich; levamisole was obtained from Solarbio. Molecular biology reagents were purchased from GenStar, Takara (Taq DNA polymerase and PrimeSTAR Max DNA polymerase, Beijing Kangrun Chengye Biotechnology Co., Ltd., Beijing, China), TIANGEN (RNA extraction kit Takara Biomedical Technology (Beijing) Co., Ltd., Beijing, China,), and Vazyme (HiScript^®^ III RT SuperMix and 2 × ChamQ SYBR Master Mix, Vazyme Biotech Co., Ltd., Nanjing, China). Key equipment included a Motic SMZ-168 stereomicroscope (Motic China Group Co., Ltd., Xiamen, China), a Leica DM6B fluorescence microscope (Leica Microsystems CMS GmbH, Wetzlar, Germany), a GeneExplorer thermal cycler (Hangzhou Bioer Technology Co., Ltd., Hangzhou, China), a Roche LightCycler^®^ 96 real-time PCR system (Roche Diagnostics GmbH, Mannheim, Germany), a DYY-6D electrophoresis unit (Beijing Liuyi Biotechnology Co., Ltd., Beijing, China), an H3-20KR refrigerated centrifuge (Hunan Kecheng Instrument Equipment Co., Ltd., Changsha, China), and a −80 °C ultra-low freezer (Qingdao Haier Biomedical Co., Ltd., Qingdao, China). The wild-type strain was N2 (Bristol); CB4856 (Hawaiian) was used for SNP mapping. The *nse-1::gfp* reporter was generated in-house by microinjection in the N2 strain. DNA repair pathway control mutants (*mus-81*, *brc-1*, and *lig-4*) were maintained under the same standard conditions as other worm strains, namely at 20 °C on NGM agar plates seeded with *Escherichia coli* OP50, and M9 buffer was used for washing, transfer, and treatments.

### 4.2. Worm Culture and Maintenance

A transgenic *nse-1::gfp* reporter strain, previously generated in our laboratory, was used throughout this study [12]. The reporter expresses an NSE-1–GFP fusion protein in an otherwise wild-type background to enable visualization of NSE-1 subcellular localization; unless otherwise stated, all mutant strains were generated and analyzed in the *nse-1::gfp* background. Worms were maintained at 20 °C on NGM agar plates seeded with *Escherichia coli* OP50, and M9 buffer was used for washing, transfer, and treatments [30]. When synchronization was required, embryos were obtained either by alkaline hypochlorite bleaching or by egg laying from gravid adults, and were hatched overnight in M9 without food to obtain synchronized L1 larvae. Developmental stages were assigned based on body size and morphological criteria, and stage-matched animals were used in all assays to minimize the confounding effects of developmental variation.

### 4.3. nse-1::gfp Reporter and Localization Scoring

In the *nse-1::gfp* reporter strain, NSE-1::GFP is predominantly nuclear under normal conditions and displays a relatively stable intranuclear distribution. Because NSE-1 is an essential subunit of the SMC-5/6 complex and its localization depends on complex integrity and functional status, NSE-1::GFP nuclear localization was used as a phenotypic readout to establish the screening platform for identifying SMC-5/6 dysfunction. For screening and subsequent phenotypic analyses, stage-matched animals were selected. After mounting, images of gonadal cells were acquired by fluorescence microscopy, using the stable nuclear enrichment pattern observed in the *nse-1::gfp* control as the baseline for normal localization. All samples were imaged using identical objectives and acquisition settings to ensure comparability. Aberrant localization was scored when nuclear enrichment was markedly reduced and accompanied by a diffuse signal.

### 4.4. EMS-Based Forward Screen and Mutant Line Establishment

An EMS-based forward genetic screen was performed in the *nse-1::gfp* reporter background to identify genetic factors affecting NSE-1 subcellular localization. The workflow comprised chemical mutagenesis, phenotypic screening, and outcrossing to reduce background mutations. Synchronized L1 larvae were cultured on OP50-seeded NGM plates at 20 °C until the L4 stage, washed from plates with M9 buffer, and collected by gentle centrifugation. Worms were resuspended in 2 mL M9, EMS was added to a final concentration of 50 mM, and animals were incubated at 20 °C for 4 h. All EMS handling was performed in a chemical fume hood with appropriate personal protective equipment, and EMS-contaminated waste was collected and disposed of in accordance with institutional safety guidelines. Mutagenesis was terminated by washing twice with M9 to remove residual EMS, followed by recovery on fresh OP50-seeded NGM plates.

P0 animals were allowed to self-fertilize to generate F1 progeny, and F1 animals were selfed to obtain F2. Individual F2 animals were singled to establish independent lines; when the progeny reached the F3 adult stage, NSE-1::GFP localization in gonadal cells was examined under standardized imaging conditions to identify candidate mutants showing reproducible mislocalization.

Each candidate line was outcrossed to males of the parental *nse-1::gfp* strain to reduce EMS-induced background mutations. Young adult hermaphrodites were used as mothers and were mated with males for 12 h, after which individual animals were singled to plates. Successful crosses were inferred from an increased proportion of males among the progeny. In each subsequent generation, lines were selected that retained the NSE-1::GFP mislocalization phenotype while maintaining the reporter background. Outcrossing was repeated for four generations to obtain stable homozygous mutant strains for genetic mapping and phenotypic analyses.

### 4.5. SNP Mapping and Whole-Genome Sequencing

Stable homozygous mutant strains were subjected to SNP-based chromosomal mapping and interval mapping, followed by whole-genome sequencing (WGS) to systematically pinpoint the causal lesion. Initial chromosomal assignment was performed using the 48-marker SNP mapping strategy described by Davis et al. [31], which distinguishes the N2 (Bristol) strain from the CB4856 (Hawaiian) isolate. Homozygous mutants were crossed with CB4856, and 20 F1 L4 animals were singled from plates with ~50% (indicative of successful crosses). After F1 self-fertilization, approximately 400 F2 L4 animals were randomly selected and propagated. When their progeny reached the F3 generation, animals were scored based on the NSE-1::GFP phenotype. For each mutant, 40–50 F3 individuals exhibiting the mutant phenotype and an equivalent number of phenotypically normal controls were collected. Animals were lysed, and samples with the same phenotypic class were pooled to generate PCR templates (phenotype-based pooling). SNP markers on each chromosome were genotyped by PCR amplification followed by DraI restriction digestion and agarose gel electrophoresis (PCR–RFLP). Recombination patterns were compared between the mutant-phenotype pool and the control pool. Enrichment of the N2 allele at markers within a chromosomal region in the mutant-phenotype pool was taken as evidence of linkage, thereby assigning the mutation to the corresponding chromosome. After chromosomal assignment, interval mapping was performed to refine the locus. Forty to fifty F3 plates showing either the mutant phenotype or segregation of the phenotype in the CB4856 background were selected. From each plate, five worms were lysed to prepare an individual PCR template (one plate per tube). Multiple SNP markers spanning the candidate chromosome were then genotyped by PCR–RFLP (PCR amplification followed by DraI digestion and gel electrophoresis). By comparing recombination events across markers, the linked interval was progressively narrowed to fine-map the mutation. Following genetic mapping, mutant strains and the parental *nse-1::gfp* control were expanded in parallel, and ~3 × 10^4^ worms were collected per sample.

Worms were washed repeatedly with M9 buffer to minimize OP50 bacterial contamination, pelleted, and snap-frozen in liquid nitrogen. Samples were submitted to Shanghai Institute for Biomedical and Pharmaceutical Technologies for whole-genome sequencing (WGS). Sequencing libraries were prepared using a standard Illumina paired-end DNA library protocol with an average insert size of approximately 350 bp and sequenced on an Illumina HiSeq X10 platform in paired-end 150 bp (PE150) mode, with a target coverage depth of at least 30× per sample. Raw reads were filtered to remove adapter-contaminated reads, reads containing more than 10% ambiguous bases (N), and low-quality reads in which more than 50% of bases had a Phred quality score ≤20. Clean reads were aligned to the *nse-1::gfp* reference genome using BWA-MEM. PCR duplicates were marked using Picard, and alignment files were processed with SAMtools. SNPs and small indels were identified using a standard variant-calling pipeline based on GATK HaplotypeCaller. Variants detected in each mutant were compared against those in the parental *nse-1::gfp* strain to identify mutant-specific variants. Candidate causal mutations were prioritized by integrating WGS calls with the chromosomal and interval mapping results, ultimately identifying the genes responsible for NSE-1::GFP mislocalization.

### 4.6. Fertility and Genotoxin Sensitivity Assays

Reproductive traits and DNA damage sensitivity were examined in mutant and control animals to assess phenotypic changes in reproductive development and DNA damage responses. All assays were performed using stage-matched worms and were repeated at least three times. OP50 was diluted in M9 buffer to facilitate accurate egg counting. For each strain, 30 L4-stage worms were singled onto plates seeded with diluted OP50 and maintained at 20 °C. Animals were transferred to fresh plates every 12 h, and the total number of eggs laid on the previous plate was counted immediately after transfer until egg laying ceased. Unhatched embryos were counted the following day, and progeny viability (hatching rate) was calculated as the number of hatched larvae divided by the total number of eggs laid. Male progeny were scored 3 days later, and the male frequency was calculated as the percentage of males among surviving animals. To apply distinct genotoxic stress conditions, cisplatin, methyl methanesulfonate (MMS), and hydroxyurea (HU) were diluted in M9 buffer and mixed with 5% concentrated OP50. L1- and L4-stage animals were exposed to the indicated agents under these conditions. Cisplatin and MMS treatments were performed for 16 h, whereas HU treatment was performed for 20 h. After drug exposure, animals were washed to remove residual compounds, and progeny hatching rates and the proportions of animals at each developmental stage were quantified.

### 4.7. Structural Modeling and Analysis

A homology model of *C. elegans* SMC-6 was built using SWISS-MODEL with the cryo-EM structure of the *Saccharomyces cerevisiae* SMC-5/6 complex as a template (PDB: 7Y8L). Mutations (Q69*, R103*, and P514L) were introduced and visualized in PyMOL v2.5. Structural alignments and analyses of domain–domain interactions were performed using ChimeraX v1.6. Differences in hydrogen-bonding networks and electrostatic interactions between the wild-type and mutant models were evaluated.

### 4.8. Statistical Analysis

All experiments were independently repeated at least three times. Data are shown as mean ± SEM. Statistical analyses were performed using GraphPad Prism 10. One-way ANOVA with Tukey’s multiple-comparisons test was used for analyses involving more than two groups, whereas two-tailed Student’s *t* tests were used for pairwise comparisons. Statistical significance was defined as *p* < 0.05.

## 5. Conclusions

We established an experimentally tractable and scalable visualization-based forward genetic screening platform by using germline nuclear localization of NSE-1::GFP as an in vivo readout of the functional state of the SMC-5/6 complex. By screening ~26,600 EMS-mutagenized genomes, we isolated heritable mutants exhibiting aberrant NSE-1::GFP localization and identified three independent *smc-6* alleles (*wsh34*, *wsh35*, and *wsh36*), thereby generating an *smc-6* allelic series for structure–function analyses. Molecular characterization and domain mapping revealed that two nonsense mutations are located in the ATPase head, whereas the missense mutation affects a highly conserved site in the hinge region. Consistent with these molecular lesions, the alleles displayed concordant stratified differences in fertility/progeny viability, sensitivity to DNA-damaging agents (MMS, HU, and cisplatin), developmental delay, and induction of apoptosis marker genes (*egl-1* and *ced-13*), supporting a domain-hierarchy model in which the ATPase head is essential, whereas the hinge contributes to structural regulation and robustness. Together with structural modeling, we propose that ATPase-head integrity is critical for complex assembly/conformational transitions and chromatin-associated functions, whereas hinge perturbation is more likely to compromise conformational flexibility and dynamics, resulting in partial functional defects under replication stress and specific damage contexts. Overall, this study not only provides new genetic tools and phenotypic resources for *smc-6* loss-of-function analysis but also demonstrates that NSE-1::GFP localization is an effective entry point for dissecting SMC-5/6-mediated genome maintenance in vivo. Future work combining allelic rescue/CRISPR recapitulation, direct measurements of complex assembly and chromatin binding, and genetic interaction analyses with key DNA repair factors will further clarify the precise roles of SMC-5/6 in replication-stress responses and germline genome stability.

## Data Availability

The original contributions presented in this study are included in the article. Further inquiries can be directed to the corresponding authors.

## Abbreviations

SMC, Structural Maintenance of Chromosomes; SMC-5/6, Structural Maintenance of Chromosomes 5/6; NSE, Non-SMC Element; GFP, Green Fluorescent Protein; EMS, Ethyl Methanesulfonate; SNP, Single-Nucleotide Polymorphism; WGS, Whole-Genome Sequencing; DNA, Deoxyribonucleic Acid; RNA, Ribonucleic Acid; mRNA, Messenger RNA; ATPase, Adenosine Triphosphatase; NTPase, Nucleoside Triphosphatase; MMS, Methyl Methanesulfonate; HU, Hydroxyurea; RT-qPCR, Reverse Transcription Quantitative Polymerase Chain Reaction; CEP-1, *C. elegans* p53 Ortholog CEP-1; p53, Tumor Protein p53; PCR, Polymerase Chain Reaction; RFLP, Restriction Fragment Length Polymorphism; LG, Linkage Group; cM, Centimorgan; NGM, Nematode Growth Medium; PE150, Paired-End 150 bp; bp, Base Pair; BWA-MEM, Burrows–Wheeler Aligner Maximal Exact Matches; SAMtools, Sequence Alignment/Map Tools; GATK, Genome Analysis Toolkit; ChIP, Chromatin Immunoprecipitation; cryo-EM, Cryo-Electron Microscopy; PDB, Protein Data Bank; SEM, Standard Error of the Mean; ANOVA, Analysis of Variance; PPE, Personal Protective Equipment; P0, parental generation; F1–F3, filial generations; L1–L4, larval stages 1–4.
